# Incorporating Technology in Pharmacy Education: Students' Preferences and Learning Outcomes

**DOI:** 10.7759/cureus.50158

**Published:** 2023-12-08

**Authors:** Anas Alhur, Remas Hedesh, Mara Alshehri, Shaima Al Qasim, Roaa Alkhaldi, Walaa Bazuhair, Wafa Bin Shamlan, Shatha Alshahrani, Shahad Alshahrani, Alaa Alasiri, Rahaf Alshalwi, Sara Alnefaie, Rana Alotaibi, Ragad K Aljehani, Laila Alzahrani

**Affiliations:** 1 Health Sciences, Medicine, University of Hail, Hail, SAU; 2 Pharmacy, King Khalid University, Abha, SAU; 3 Pharmacy, Taif University, Taif, SAU; 4 Medical Studies, Ibn Sina National College, Jeddah, SAU; 5 Pharmacy, Al Murjan Hospital, Jeddah, SAU; 6 Community Pharmacy, Aljamaa Pharmacy, Dammam, SAU; 7 Pharmacy, King Saud University, Riyadh, SAU

**Keywords:** learning experiences, learning outcomes, students' preferences, pharmacy education, technology

## Abstract

Introduction

The integration of technology in pharmacy education has led to significant changes, making it essential to examine its impact on student learning outcomes. With the rise of emerging technologies, such as game-based learning and augmented reality, new pedagogical approaches have been introduced. However, these technologies also bring challenges that necessitate well-thought-out responses from educational institutions, especially considering the effects of the COVID-19 pandemic on learning formats.

Methods

A cross-sectional study was conducted with pharmacy students in the Kingdom of Saudi Arabia, utilizing stratified random sampling for data collection. The study employed a validated questionnaire to gather information on students' use of technology, their preferences, and their perceived learning outcomes. The analysis was carried out using Statistical Product and Service Solutions (SPSS, version 29) (IBM SPSS Statistics for Windows, Armonk, NY), ensuring adherence to the ethical standards set by the institutional review board.

Results

The study, involving 508 pharmacy students from Saudi Arabia, revealed that a significant majority regularly use technology in their education, particularly online learning management systems and virtual labs, indicating a shift toward more accessible and efficient learning methods. A notable portion of the students acknowledged the beneficial impact of technology on their learning, with 86.3% reporting improved knowledge retention, 92% noting enhanced skills, and 87.1% observing better academic performance. However, the study also uncovered challenges, with about half of the students facing issues mainly related to distractions while finding technological complexity less problematic. The support from educational institutions in addressing these issues was found to be moderate. These findings underscore the crucial role of technology in enhancing pharmacy education while highlighting the need for more comprehensive institutional support and strategic planning to address the challenges faced by students.

Conclusions

Technology plays a vital role in the modernization of pharmacy education, significantly aiding in knowledge retention, skill development, and academic performance. Despite the widespread use of technology, the varied experiences and challenges encountered by students underscore the need for increased institutional support and strategic planning. Education must adapt alongside technological advancements to cater to the diverse needs of learners, highlighting the importance of further research into the long-term effects and optimal strategies for technology integration in pharmacy education.

## Introduction

The integration of technology into education has brought about transformative changes in learning methodologies and student engagement, with pharmacy education being no exception. The introduction of digital tools and platforms has catalyzed a shift in the educational paradigm, offering both opportunities and challenges for curriculum development and instructional practices. As the educational landscape progresses, it is critical to understand the integration of these technological advances into pharmacy curricula and their subsequent effects on student learning outcomes.

In pharmacy education, emerging technologies, such as game-based learning (GBL) and augmented reality (AR), are increasingly influential in shaping teaching approaches. These technologies range from complex simulations to basic interactive models, potentially boosting student engagement and facilitating knowledge acquisition [1,2]. Moreover, in 2023, Alhur et al. provided empirical evidence suggesting that the incorporation of health information technology into the pharmacy curriculum is considered essential for the evolving role of pharmacists, as supported by 88% of surveyed pharmacy students [3]. Despite their benefits, adopting these innovations faces challenges, such as financial constraints, technological limitations, and the need for effective assessment methods to gauge learning outcomes in environments rich with technology [1].

Educational modalities notably shifted due to the coronavirus disease 2019 (COVID-19) pandemic, leading to research on the psychological impacts of transitioning from traditional to online and blended learning environments [4-6]. These studies reveal various student preferences and highlight the significance of accommodating different learning styles in educational strategy development and implementation [7]. Our study aimed to critically assess the current state of technology integration in pharmacy education and its impact on academic performance, emphasizing student preferences and the effectiveness of such technologies.

## Materials and methods

Research design

We conducted a cross-sectional study in which data were collected from multiple participants over one week. This design was selected for its effectiveness in efficiently acquiring data from a broad sample and is well-suited for evaluating the present integration of technology in pharmacy education.

Population and sample

The target population for this study comprised pharmacy students from various institutions across the Kingdom of Saudi Arabia (KSA). To achieve a representative sample reflective of this diverse group, we employed a stratified random sampling method. This approach was designed to ensure a balanced representation across different academic years, genders, and educational institutions.

Our inclusion criteria were quite specific: the study focused on pharmacy students within KSA who actively engaged with technology in their educational pursuits. This criterion was pivotal in maintaining the relevance and focus of the research, centering on individuals directly involved in technology-enhanced learning within the pharmacy field.

Conversely, the exclusion criteria were set to omit individuals not fitting this profile. This included non-pharmacy students in KSA, pharmacy students who did not actively utilize technology in their education, and those who did not provide informed consent. Additionally, students from academic institutions that fell outside the scope of this study were excluded. This exclusion ensured that the sample remained strictly pertinent to the study's objectives, concentrating solely on pharmacy students immersed in technology-enhanced learning environments.

In total, 508 consenting students formed the sample for this study. This sample size was determined through a statistical power analysis, ensuring adequate representation and validity of the findings.

By adopting this sampling strategy, the study aimed to address the broader challenge of diversity in healthcare research. While focusing on variables such as academic year, gender, and institution, it is crucial to note that diversity in research encompasses additional dimensions, such as socioeconomic backgrounds, cultural and ethnic differences, geographical locations, and individual learning needs, including those with disabilities. Considering these factors is essential for a comprehensive understanding of the impact of technology in pharmacy education and for ensuring that research outcomes are applicable and beneficial to all segments of the student population.

Data collection

We used a validated questionnaire previously established by researchers in 2008 and 2016 [7]. The structured questionnaire included Likert scale items to measure students’ preferences and perceptions of technology in their education, multiple-choice questions to determine the types of technology tools and platforms students are acquainted with, and open-ended questions to elicit further insights or comments from the participants.

The questionnaire was distributed electronically across various social media platforms, with a completion window of one week. Consent was acquired electronically at the survey’s outset, ensuring participants were informed about the study’s aims and their rights.

Data analysis

The data were analyzed with Statistical Products and Service Solutions (SPSS) for Windows, version 29 (IBM Corp., Armonk, NY). We employed descriptive statistics such as mean, median, mode, and standard deviation to summarize the findings.

Validity and reliability

To ensure the reliability of the questionnaire domains, we conducted a preliminary analysis to calculate Cronbach’s alpha for each set of items. The domains assessed included the current state of technology integration, student preferences, perceived learning outcomes, and the barriers and challenges to technology use. Cronbach’s alpha for these domains ranged from 0.773 to 0.885, indicating an acceptable to a good level of internal consistency. Details of these reliability statistics are presented in Table 1.

**Table 1:** Reliability of questionnaire domains assessing pharmacy education technology integration

No	Variables	Cronbach’s Alpha	Number of Items
1	Current State of Technology Integration	0.773	2
2	Student Preferences	0.885	2
3	Learning Outcomes	0.775	2
4	Barriers and Challenges	0.826	3

Ethical considerations

Participants were informed of the study’s objectives, their rights, and the confidentiality of their responses. Participation was entirely voluntary, and they had the right to withdraw at any time without consequences. The institutional review board of the University of Hail approved the study design (IRB Approval No. H-2023-194).

## Results

A total of 508 students (98.10%) consented to partake in the survey. Of the 508 participants, most were women (n=367; 70.80%), while men accounted for 27.20% (n=141). Participants were drawn from a range of institutions, with the majority (n=140) hailing from King Khalid University (27%) and the University of Hail (n=117; 22.6%). The study encompassed individuals across all years of pharmacy education, with the largest group being graduates at 38.4% (n=199) and fifth-year students (n=79), forming 15.3% of the cohort (Table 2).

**Table 2:** Demographics and academic characteristics of pharmacy student participants

Category	Response/Option	Frequency	Percent	Mean	Std. Deviation
Participation Agreement	Agree	508	98.10%	1.0193	0.13773
	Disagree	10	1.90%		
Gender	Male	141	27.20%	1.7224	0.44824
	Female	367	70.80%		
Study Institution	King Saud University	24	4.60%	4.7745	1.35248
	Taif University	24	4.60%		
	Jazan University	4	0.80%		
	King Khalid University	140	27.00%		
	University of Hail	117	22.60%		
	Other	201	38.80%		
Study Year	First-Year Pharmacy Student	75	14.50%	4.2244	1.8549
	Second-Year Pharmacy Student	41	7.90%		
	Third-Year Pharmacy Student	56	10.80%		
	Fourth-Year Pharmacy Student	58	11.20%		
	Fifth-Year Pharmacy Student	79	15.30%		
	Graduate	199	38.40%		

**Figure 1:**
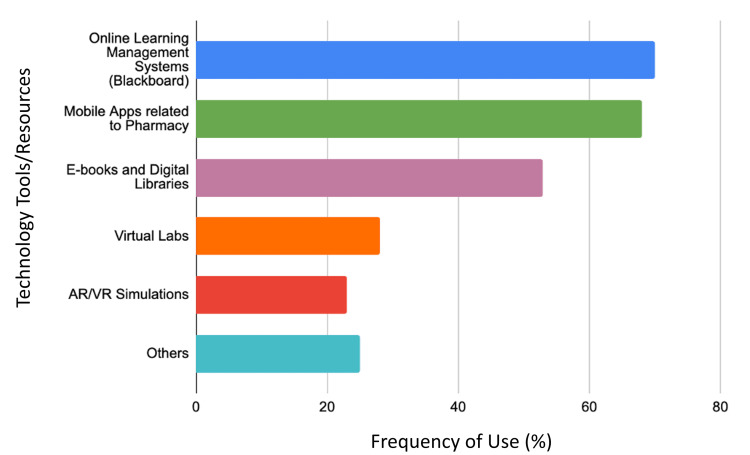
Frequency of technology tools/resources used by pharmacy students Abbreviations: AR, augmented reality; VR, virtual reality

When exploring the frequency of technology use in pharmacy education, most students frequently utilized technology, with 49.4% (n=256) always incorporating it and 34.7% (n=180) often doing so (Table 3). Approximately 70% of respondents routinely utilized online learning management systems, and approximately 65% frequently used educational apps (Figure 1). However, AR and virtual reality simulations were less commonly employed, with under 20% of participants using them frequently.

**Table 3:** Frequency of technology usage by pharmacy students in educational settings

Usage Frequency	Frequency	Percent	Mean	Std. Deviation
Always	256	49.40%	1.7008	0.87539
Often	180	34.70%		
Sometimes	45	8.70%		
Rarely	22	4.20%		
Never	5	1.00%		
Total	508	100.00%		

Concerning the comfort level with technology, many students reported a high level of ease, with 233 respondents (45%) indicating the highest comfort level on a five-point scale (Table 4). The mean comfort score was 3.9921, signifying a generally high level of comfort with technology among participants. Preferred technological tools and platforms varied, with online learning management systems being the top preference for 133 students (25.7%). Virtual Labs were also favored, selected by 91 participants (17.6%).

**Table 4:** Pharmacy students’ comfort with technology and preferences for educational tools

Question/Scale	Response/Option	Frequency	Percent	Mean	Std. Deviation
Comfort with Technology (1-5)	1 (not comfortable at all)	30	5.80%	3.9921	1.17667
	2	26	5.00%		
	3	95	18.30%		
	4	124	23.90%		
	5 (very comfortable)	233	45.00%		
Preferred Technological Tool/Platform	Online Learning Management Systems	133	25.70%	2.7382	1.40314
	Virtual Labs	91	17.60%		

Table 5 shows the results regarding integrating technology’s influence on learning outcomes. A substantial 438 respondents (86.3%) agreed that technology enhances knowledge retention. Moreover, 467 respondents (92%) confirmed that technology enhanced their skills. Additionally, 442 respondents (87.1%) attributed their improved academic performance to technology use.

**Table 5:** Perceived impact of technology integration on learning outcomes among pharmacy students

Question/Scale	Response/Option	Frequency	Percent	Mean	Std. Deviation
Integration of Technology & Knowledge Retention	Strongly agree	242	46.70%	1.685	0.80139
	Agree	205	39.60%		
	Neutral	45	8.70%		
	Disagree	11	2.10%		
	Strongly disagree	5	1.00%		
Impact of Technology on Skill Acquisition	Significantly Improved	284	54.80%	1.5138	0.64497
	Somewhat Improved	192	37.10%		
	No Impact	27	5.20%		
	Hindered	5	1.00%		
	Significantly Hindered	0	0%		
Academic Performance with Technology Integration	Much Better	259	50.00%	1.6024	0.6818
	Better	192	37.10%		
	About the Same	57	11.00%		
Total		508	100.00%		

The study also examined students’ challenges with technology in their education and the level of institutional support received. The findings indicated a dichotomous experience, with 255 respondents (49.2%) encountering challenges and 253 respondents (48.8%) not facing significant issues, indicating a need for additional research to understand and mitigate these difficulties (Table 6).

**Table 6:** Challenges and institutional support related to technology use in pharmacy education

Question/Scale	Response/Option	Frequency	Percent	Mean	Std. Deviation
Challenges in Using Technology	Yes	255	49.20%	1.498	0.50049
	No	253	48.80%		
Institutional Support (1-5)	1 (not supportive at all)	38	7.30%	3.2618	1.14284
	2	75	14.50%		
	3	204	39.40%		
	4	98	18.90%		
	5 (very supportive)	93	18.00%		
Totals		508	100.00%		

**Figure 2:**
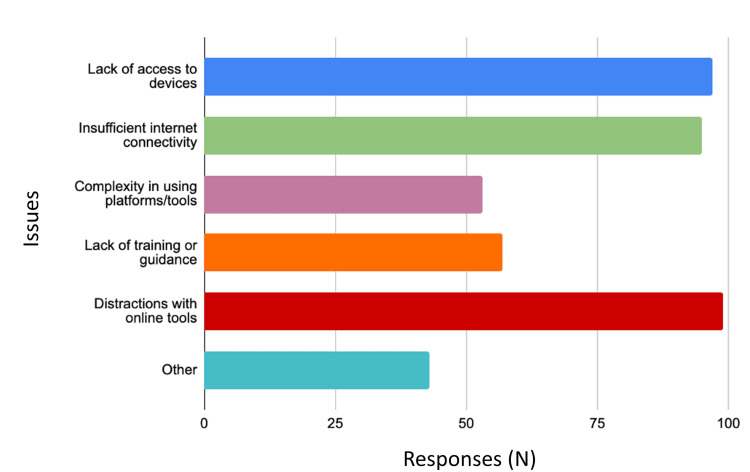
Frequency of technology issues faced by pharmacy students

In terms of institutional support, only 38 respondents (7.30%) felt unsupported, 93 (18.00%) felt very supported, and the remainder felt moderately supported, suggesting room for improvement in institutional backing for technology use. Distractions were identified as the most common issue, whereas the complexity of technology posed the least concern among the challenges reported (Figure 2).

## Discussion

This study provides insight into the incorporation of technology in pharmacy education, revealing its profound impact on students’ learning outcomes and preferences. Here, we discuss these findings within the context of existing literature and explore their broader implications.

Game-based learning

Integrating GBL in pharmacy education appears to enhance student engagement and knowledge retention. Our findings resonate with Huda et al., who report varied sophistication levels in GBL methods, from basic to advanced technological interactions [8]. Like Dabbous et al., our study suggests that GBL can positively influence learning outcomes, fostering a self-motivated and active learning environment [5]. However, financial and technological barriers and a lack of rigorous assessment methods require further attention.

AR in education

Our study aligns with the work of Dhar et al., suggesting that AR offers unique learning advantages, such as remote learning capabilities and interactive simulations, potentially revolutionizing pharmacy education [6]. Despite its potential, the full integration of AR into the curriculum is challenged by the need for continuous innovation and research to overcome existing barriers.

Impact of COVID-19 on education modalities

The COVID-19 pandemic necessitated a pivot to online learning, with mixed reactions from students. Basheti et al. found a preference for traditional learning despite the perceived stress reduction associated with online formats [2]. Additionally, Dabbous et al. identified concerns about the long-term efficacy of online learning on student performance [5]. These findings underscore the need for balanced, flexible learning strategies to adapt to such unprecedented challenges.

Learning preferences and styles

Our study’s indication of a preference for aural and kinesthetic learning styles among pharmacy students suggests a need for educational strategies that cater to these preferences [7]. Understanding these learning styles is crucial for educators to tailor their methods and maximize educational outcomes.

Simulation in education

The application of simulation in pharmacy education offers a safe space for skill practice [9]. McBane et al. highlighted the diverse potential of simulation techniques, yet they also noted limited research on the direct benefits for learning outcomes [10]. This gap suggests an opportunity for future research to validate the effectiveness of simulation-based education.

Students’ technology use and perception

The high percentage of pharmacy students incorporating technology in their education and their comfort in using online learning management systems aligns with that of Medina et al., who highlighted these systems’ benefits for interactive learning [11]. Our findings further suggest that technology has positively impacted knowledge retention and skill acquisition, corroborated by Müller et al. [12] and Cooper et al. [4], who also acknowledge technology’s role in enhancing student performance.

Challenges and institutional support

Despite the benefits, our study revealed a divided opinion on technology integration challenges. The moderate level of institutional support observed indicates substantial room for improvement in this domain. Future research should prioritize identifying and overcoming these hurdles to optimize the benefits of technology in education.

Limitations

This study offers important insights but also presents several limitations. Firstly, its focus on pharmacy colleges within the KSA limits the generalizability of its findings, as these may not be representative of students in different geographic or cultural contexts. Additionally, its cross-sectional nature only provides a snapshot in time, lacking the ability to track changes or establish causality. The reliance on self-reported questionnaires could lead to response bias, with participants potentially providing socially desirable answers or inaccurately recalling their experiences. Furthermore, the study's emphasis on quantitative data might have restricted the depth of exploration into students' experiences, which could have been enriched by qualitative insights such as interviews or open-ended questions. Other factors, such as individual learning styles, technological competencies, or variations in institutional technological infrastructure, may not have been fully explored. Recognizing these limitations is crucial, especially as the study highlights the widespread use of technology in education and its impact on learning outcomes. This underscores the need for educational strategies and institutional support systems that evolve with technological advancements. Future research should aim to understand the long-term effects of technology integration in pharmacy education and develop comprehensive strategies that maximize its potential, catering to the diverse needs of learners for more inclusive and effective educational experiences.

## Conclusions

This research highlights the transformative impact of technology in the realm of pharmacy education, notably enhancing learning experiences and fostering improvements in areas such as knowledge retention, skill development, and overall academic performance. The widespread adoption of technology among the student population emphasizes its critical role in the contemporary educational framework. Nonetheless, the variability in student experiences and the obstacles faced in technology integration underscores the necessity for substantial institutional support and strategic planning. As the educational environment continually adapts to technological advancements, it becomes imperative for academic institutions to address these challenges effectively. Doing so ensures that the incorporation of technology into the educational process is seamless, efficient, and attuned to the varied needs of students, thus maintaining the relevance and efficacy of educational methodologies in an increasingly technology-driven landscape.

## Figures and Tables

**Figure 3:**
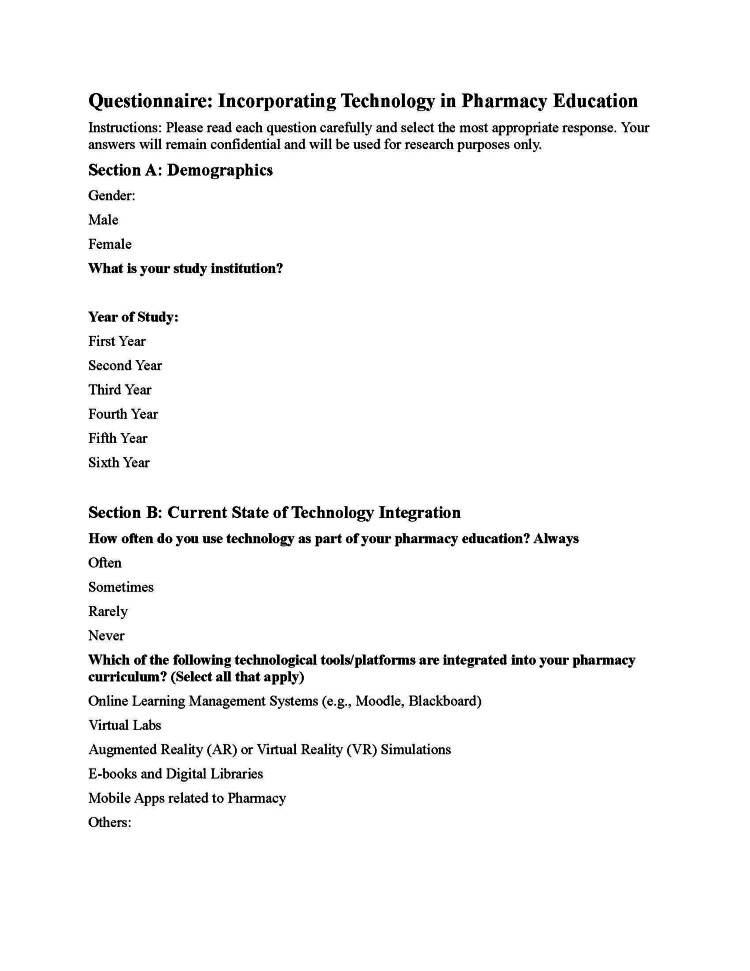
Questionnaire: incorporating technology in pharmacy education

**Figure 4:**
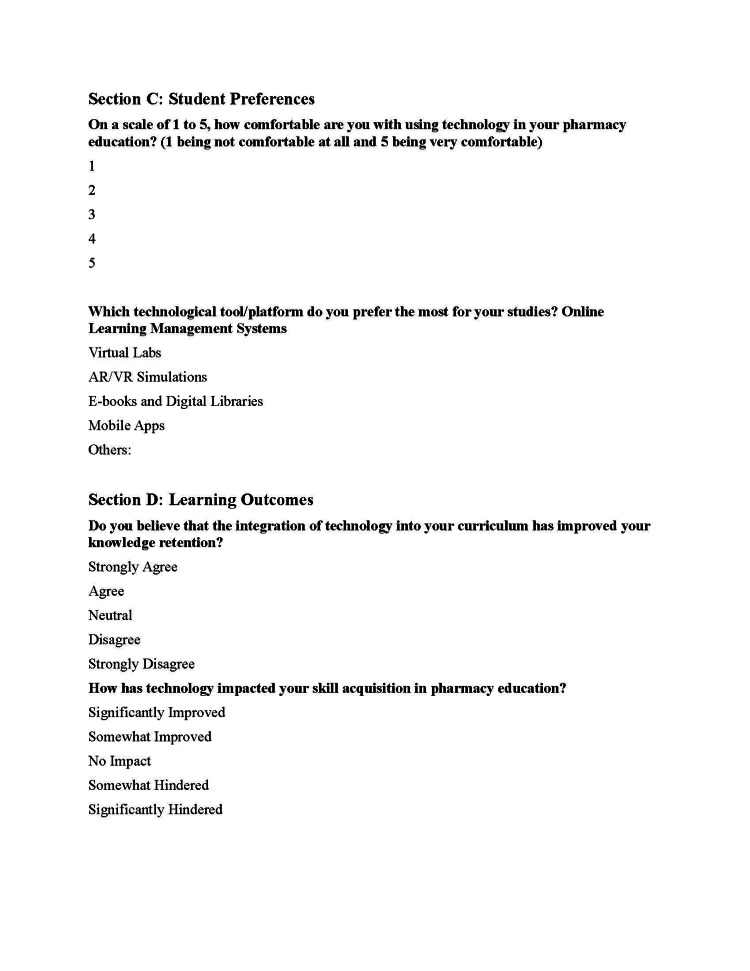
Questionnaire: incorporating technology in pharmacy education

**Figure 5:**
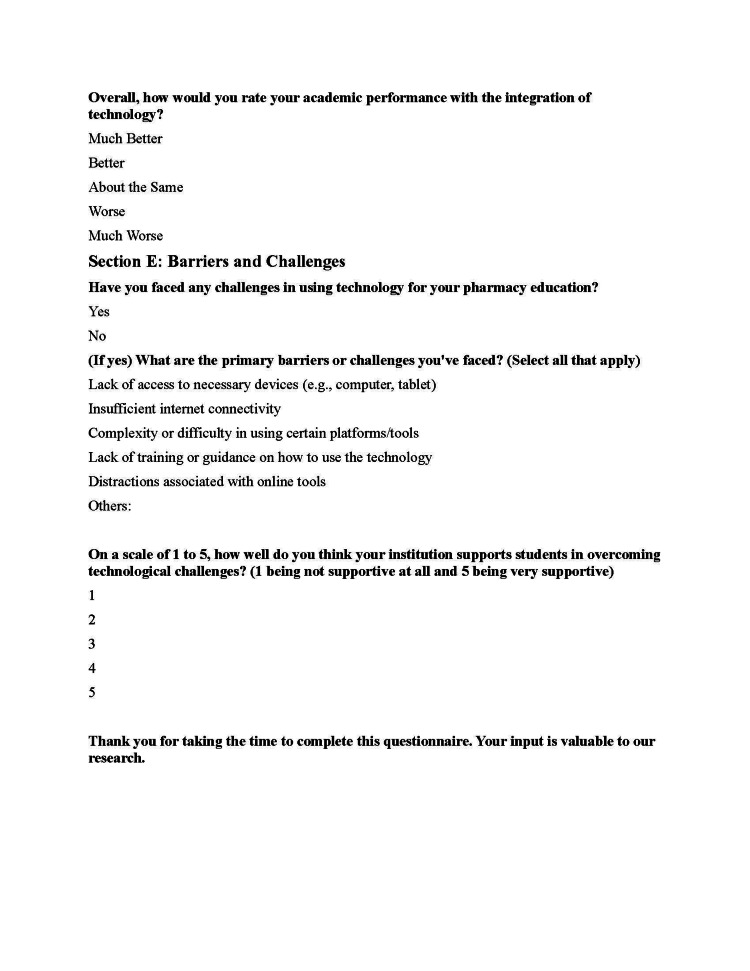
Questionnaire: incorporating technology in pharmacy education
